# Nuclear localization of the CK2α-subunit correlates with poor prognosis in clear cell renal cell carcinoma

**DOI:** 10.18632/oncotarget.13693

**Published:** 2016-11-29

**Authors:** Maj Rabjerg, Barbara Guerra, Aida Oliván-Viguera, Minne Line Nedergaard Mikkelsen, Ralf Köhler, Olaf-Georg Issinger, Niels Marcussen

**Affiliations:** ^1^ Department of Pathology, Odense University Hospital, DK-5000 Odense, Denmark; ^2^ Department of Biochemistry and Molecular Biology, University of Southern Denmark, DK-5230 Odense, Denmark; ^3^ Aragon Agency for Research and Development (ARAID), IACS, IIS Aragon, 50009 Zaragoza, Spain

**Keywords:** renal cancer, protein kinase CK2, CK2 subunits, CK2-targeted therapy, CX-4945

## Abstract

Protein kinase CK2α, one of the two catalytic isoforms of the protein kinase CK2 has been shown to contribute to tumor development, tumor proliferation and suppression of apoptosis in various malignancies. We conducted this study to investigate CK2 expression in different subtypes of Renal Cell Carcinoma (RCC) and in the benign oncocytoma. qRT-PCR, immunohistochemistry and Western blot analyses revealed that CK2α expression was significantly increased at the mRNA and protein levels in clear cell RCC (ccRCC). Also the kinase activity of CK2 was significantly increased in ccRCC compared to normal renal cortex. Nuclear protein expression of CK2α correlated in univariate analysis with poor Progression Free Survival (HR = 8.11, p = 0.016). Functional analyses (cell proliferation assay) revealed an inhibitory effect of Caki-2 cell growth following CK2 inhibition with CX-4945. Our results suggest that CK2α promotes migration and invasion of ccRCC and therefore could serve as a novel prognostic biomarker and molecular therapeutic target in this type of cancer.

## INTRODUCTION

Renal cell carcinoma (RCC) is the most lethal of the common urological cancers with 338.000 new cases and 144.000 deaths registered worldwide in 2012 [1]. RCC comprises a heterogeneous group of neoplasms, and according to WHO, it is classified into four main histological subtypes based on morphology and genetics [2]. The most frequent subtype is the clear cell renal cell carcinoma (ccRCC), which comprises 75-80% of all RCCs. The papillary carcinoma (PRCC, 10% of RCC), chromophobic carcinoma (ChRCC, 5% of RCC) and collecting duct carcinoma (2% of RCC) are the other main subtypes. The benign tumor, renal oncocytoma (RO), comprises 3-5% of all adult renal tumors [3].

Widely used clinicopathological parameters, such as TNM staging and nuclear Fuhrman grade, provide robust prognostic information. However, they cannot accurately predict a reliable outcome for RCC since similar TNM stage and nuclear grade may have very different outcomes. One third of patients has metastases at diagnosis and among those with clinically localized disease, 30-40% will develop metastases after surgery [4]. Identifying this high-risk group of RCC patients remains a challenge. RCC is often late diagnosed and has a poor response to available therapies. Therefore, there is a clear need for markers that can predict outcome in RCC patients or guide treatment decisions.

Inactivation of the VHL tumor suppressor gene is observed in all inherited forms of RCC and in many of the sporadically occurring tumors [5]. Despite the role of VHL in the hypoxia pathway of the RCC pathogenesis it can be expected that also other genetic alterations are necessary for tumor formation [6]. Accordingly, there is a very active search for new biomarkers in the field of renal oncology that have the potential to further improve diagnosis, treatment and prognosis of RCC. Despite the efforts, known biomarkers did not advance to clinical routine [7].

Protein kinase CK2 is a multifunctional, ubiquitously expressed protein kinase with a large array of more than 300 substrates. Many of these are critically involved in the regulation of cell growth, proliferation and differentiation [8–11]. These include oncogenes, transcription factors and tumor-suppressor genes as well as proteins involved in signal transduction pathways. Increasing evidence indicates that CK2 is linked to cellular transformation and cancer [12, 13]. For instance, high levels and protein kinase activity of CK2 have consistently been observed in a variety of cancers, including kidney [14], breast [15], colorectal [16], head and neck cancers [17] and glioblastoma [18]. Overexpression and prognostic significance of CK2α subunit have been observed in lung cancer, prostate cancer, and leukemia [19–21].

The kinase has a tetrameric structure of the catalytic subunits, α and α', and the regulatory β subunit with an α_2_β_2_, αα'b_2_ or α'_2_β_2_ configuration and is localized in both the nucleus and cytosolic subcellular compartments [22]. Not only the holoenzyme, but also the isolated catalytic subunits are active and can exert independent functions in cells [23].

In a previous study we could show that high mRNA expression of CK2α was associated with poor prognosis in ccRCC [24]. However, the detailed prognostic and functional role of CK2α in human RCC is still to be explored.

The aim of this study was to investigate mRNA expression of the different subunits of CK2 in ccRCC, protein levels of CK2α in different subtypes of RCC and in renal oncocytoma and to evaluate the prognostic value of protein expression of CK2α in ccRCC patients. Additionally, we aimed at verifying whether chemical inhibition of CK2 affected proliferation of human cancer cells lines in vitro.

## RESULTS

### Patient characteristics

We examined a total of 155 patients with different subtypes of RCC and oncocytoma. There were 94 males and 61 females with a mean age of 61.9 years (range 28.1-86.4). The mean tumor size was 6.73 cm (range 1.5-22). Clinical and demographic data for all patients included are summarized in Table 1.

**Table 1:** Clinical and demographic data of the 155 patients included in the TMAs

	ccRCC (n=105)	PRCC (n=27)	ChRCC (n=8)	RO (n=13)	UcRCC (n=2)
Sex					
Male	60 (57)	22 (81.5)	5 (62.5)	6 (46.2)	1 (50)
Female	45 (43)	5 (18.5)	3 (37.5)	7 (53.9)	1 (50)
Age, years	62.2 (range 28.1-86.4)	61.2 (range 28.4-81.0)	51.5 (range 38.6-63.9)	66.2 (range 39.5-81.5)	70.5 (range 69.6-71.4)
Tumor size, cm	7.2 (range 2-22)	5.0 (range 1.5-11)	7.4 (range 3.7-11.5)	5.6 (range 2.2-15)	10.5 (8-13)
pT stage		§			
pT1 a/b	43 (41)	17 (65.4)	2 (25)	.	0
pT2 a/b	22 (21)	3 (11.5)	3 (37.5)	.	1 (50)
pT3 a/b	39 (37)	5 (19.2)	3 (37.5)	.	1 (50)
pT4	1 (1)	1 (3.9)	0 (0)	.	0
Late metastasis	32 (30.5)	6 (22.2)	0 (0)	.	1 (50)
Fuhrman grade					
G1	6 (5.7)	0 (0)	0 (0)	.	0 (0)
G2	51 (48.6)	17 (63)	1 (12.5)	.	0 (0)
G3	37 (35.2)	10 (37)	4 (50)	.	2 (100)
G4	11 (10.5)	0 (0)	3 (37.5)	.	0 (0)
Leibovich score#					
0-2	30 (33)	.	.	.	.
3-5	39 (43)	.	.	.	.
≥ 6	22 (24)	.	.	.	.

§ Missing information on one patient.

# Only patients with non-metastatic RCC at the time of diagnosis was assigned a Leibovich score (n=91).

The table specifies numbers (%) for categorical variables and mean (range) for continuous variables. Abbreviations: ccRCC (clear cell Renal Cell Carcinoma), PRCC (Papillary Renal Cell Carcinoma), ChRCC (Chromophobic Renal Cell Carcinoma), RO (Renal Oncocytoma), UcRCC (Unclassifiable Renal Cell Carcinoma).

### Overexpression of CK2α in human ccRCC

**Figure 1:**
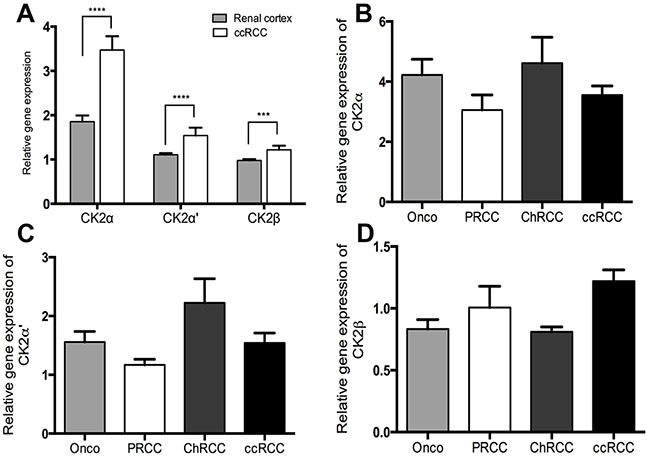
**A.** Relative gene expression levels of the CK2 subunits α, α' and β by qRT-PCR showing a significantly higher expression in ccRCC than matched normal renal cortex. **B-C.** Gene expression levels of the three CK2 subunits in different subtypes of RCC (ccRCC, clear cell renal cell carcinoma; PRCC, papillary renal cell carcinoma; ChRCC, chromophobe renal cell carcinoma) and in the benign oncocytoma (Onco). No significant difference was found.^***^ *p*< 0.001, ^****^p< 0.000.

**Figure 2:**
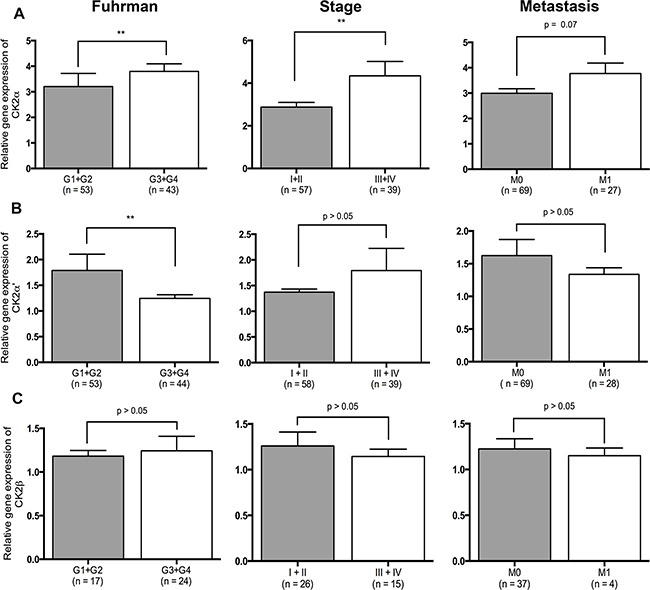
Relative gene expression levels of the three CK2 subunits and their correlation to clinicopathological factors **A.** High expression of CK2α correlates to high Fuhrman grade (*p*=0.001), high stage (*p*=0.007) and distant metastasis (*p*=0.07). **B.** High expression of CK2α' correlates to low Fuhrman grade (*p*=0.01). **C.** Expression of CK2β did not correlate to any of the clinicopathological factors.

Quantitative RT-PCR data from 97 ccRCCs were generated from another study [24]. In addition, qRT-PCR data were generated for 23 PRCC, 8 ChRCC and 11 RO for the purpose of this study. CK2α was expressed 1.87 fold higher in the tumor tissue of ccRCC than normal renal cortex (*p* < 0.0001). (Figure 1A). No statistically significant difference was observed between the different subtypes of RCC, RO and the subunits of CK2, Figure 1B-1D. The correlation of mRNA expression of the different CK2 subunits in ccRCC to Fuhrman grade, tumor stage and metastasis showed a significant higher CK2α expression in high Fuhrman grade (*p*=0.001), high tumor stage (*p*=0.007) and a tendency towards a higher rate of metastasis (*p*=0.07), Figure 2A. High mRNA expression of CK2α' correlated to a low Fuhrman grade (*p*= 0.01), Figure 2B. Messenger RNA expression of CK2β did not correlate with any of the clinicopathological factors, Figure 2C.

### Nuclear localization of CK2α correlates to metastatic rate

**Figure 3:**
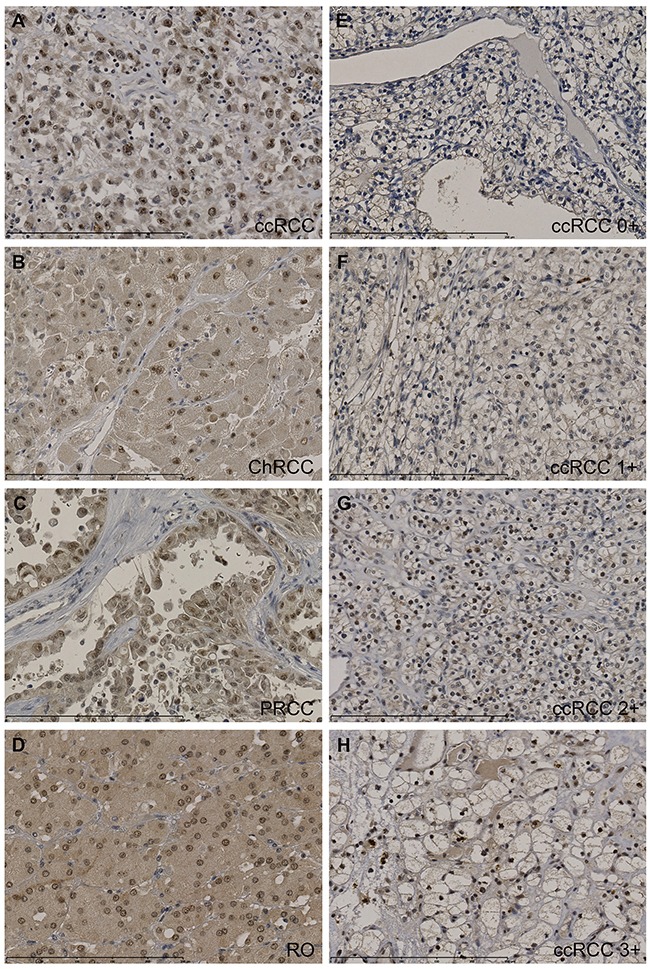
Tissue micro arrays including ccRCC (n=105), PRCC (n=27), ChRCC (n=8), UcRCC (n=2), RO (n=13) and normal renal cortex (n=146) were immunohistochemically stained for CK2α Stainings were evaluated semi-quantitatively with the following scores: the extent of staining of moderate to strong intensity in the nuclei was scored as 0 (0%), 1+ (1-25%), 2+(26-50%), 3+(51-75%) or 4+(76-100%) according to the percentages of the positive staining areas relative to the entire core area. The cytoplasm was scored as 0 or 1+, when more than 10% of the cytoplasm area showed positivity. The sum of nuclei and cytoplasm was used as the final CK2α score (0-5+). **A-D.** High protein expression of CK2α (3-5+) in ccRCC (5+), ChRCC (4+), PRCC (3+) and oncocytoma (4+). **E-H.** Representative stainings of CK2α in ccRCC. Scalebar indicates 0-250 μm. 400x magnification.

Immunohistochemistry for CK2α was carried out on all 155 cases of renal cell neoplasms using TMA. Nine cores containing normal renal cortex were lost in the processing. CK2α was strongly expressed in 19 (18%) ccRCCs, 5 PRCCs (19%), 1 ChRCC (13%) and 4 ROs (30%) (Table 2). Strong expression was defined as a score of >2+ when a sum of nuclear and cytoplasmic staining was made (Figure 3). A positive nuclear staining was found in 75% of ccRCC, 67% of PRCC, 87% of ChRCC and 92% of RO, respectively. No difference was found between normal renal cortex and ccRCC (Fisher's exact test, *p* = 0.4).

**Table 2:** Protein expression of CK2α in renal epithelial neoplasms with TMA

Renal neoplasm	CK2α (0)	CK2α (1-2+)	CK2α (3-5+)	*p*-value	CK2α Nuc-(0)	CK2α Nuc+ (1-4+)	*p*-value
ccRCC (n=105)	25 (24%)	61 (58%)	19 (18%)		26 (25%)	79 (75%)	
PRCC (n=27)	9 (33%)	13 (48%)	5 (19%)		9 (33%)	18 (67%)	
ChRCC (n=8)	1 (13%)	6 (74%)	1 (13%)		1 (13%)	7 (87%)	
RO (n=13)	1 (8%)	8 (62%)	4 (30%)		1 (8%)	12 (92%)	
UcRCC (n=2)	0 (0%)	1 (50%)	1 (50%)	0.6	0 (0%)	2 (100%)	0.4
Renal cortex (n=146)	4 (3%)	129 (88%)	13(9%)		4 (3%)	142 (97%)	

Abbreviations: CK2α, protein kinase 2 alpha subunit; TMA, tissue microarray; ccRCC, clear cell renal cell carcinoma; PRCC, papillary renal cell carcinoma; ChRCC, chromophobe renal cell carcinoma; RO, renal oncocytoma, UcRCC, unclassifiable RCC; Nuc, nuclear staining of CK2α.

**Figure 4:**
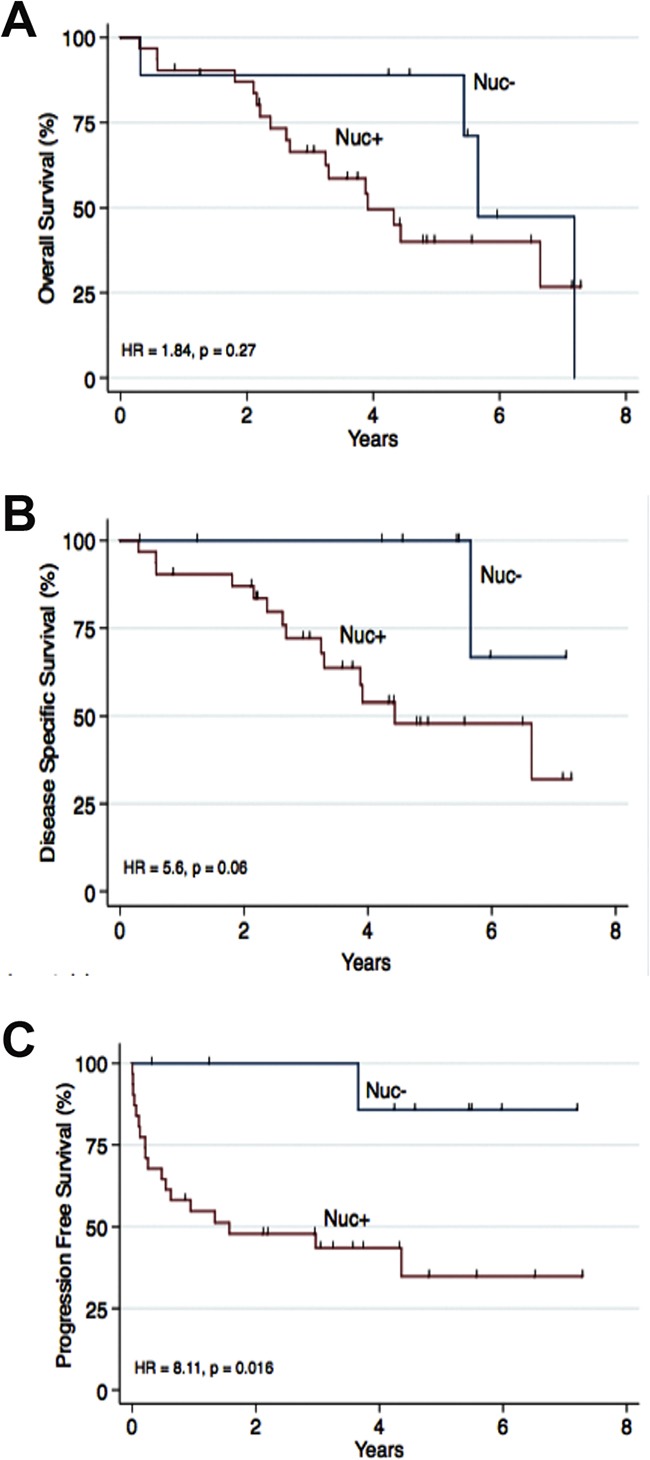
Nuclear staining of CK2α was correlated to overall survival (OS), disease specific survival (DSS) and progression free survival (PFS) in 40 patients with ccRCC high stage disease (pT3-4) Nuc- was defined as 0 (0%) positive nuclei. Nuc+ was defined as 1-4 (1-100%) positive nuclei. **A.** No association to OS was found. **B.** A tendency towards a poor DSS was seen (HR = 5.6, *p* = 0.06). **C.** A positive nuclear staining of CK2α was associated to a poor PFS (HR = 8.11, p=0.016).

The expression of CK2α in high stage ccRCC (pT3-4) was compared with gender, age, Fuhrman grade, tumor size, Leibovich score, T-stage, metastasis and VHL mutation status. A positive nuclear staining of CK2α correlated significantly with late metastasis (Table 3). We performed a Kaplan Meier survival analysis on ccRCC patients with a high pathological stage (pT3-4). There was a statistically significant correlation between the Nuc+ expression group (log-rank, *p*=0.02, HR = 8.11) and poor PFS and a tendency toward a correlation with DSS (log rank *p*=0.06, HR = 5.6) (Figure 4). In a multivariate cox regression analysis including the 40 patients with high stage ccRCC and the variables CK2α nuclear expression, Fuhrman grade G1-G2/G3-G4, tumor size <7/≥7 cm, VHL mutation and gender, nuclear expression of CK2α lost its prognostic value (Table 4).

**Table 3:** High stage ccRCC patient characteristics and protein expression of CK2α (n=40)

ccRCC	Patients (%)	CK2α (0-2+)	CK2α (3-5+)	*p*-value	Nuc −	Nuc +	*p*-value
Sex							
Male	24 (60)	21	3		8	16	
Female	16 (40)	13	3	0.59	1	15	0.044*
Age§							
≤ 64	20 (50)	16	4		5	15	
> 64	20 (50)	18	2	0.38	4	16	0.7
Fuhrman							
G1+G2	17 (42.5)	13	4		5	12	
G3+G4	23 (57.5)	21	2	0.19	4	19	0.37
Tumorsize§							
< 7	16 (40)	12	4		5	11	
≥ 7	23 (57.5)	21	2	0.17	4	19	0.31
Leibovich score#							
0-2	0 (0)	0	0		0	0	
3-5	15 (37.5)	11	4		4	11	
≥ 6	15 (37.5)	14	1	0.14	4	11	1.0
Late metastasis							
No	21 (52.5)	17	4		8	13	
Yes	19 (47.5)	17	2	0.45	1	18	0.013*
VHL mutation							
No	25 (62.5)	23	2		5	20	
Yes	15 (37.5)	11	4	0.11	4	11	0.63

§ : missing information on one patient.

* *p* <0.05.

# Only patients with non-metastatic RCC at the time of diagnosis was assigned a Leibovich score (n=30).

**Table 4:** Multivariate Cox regression analysis including clinical variables for high stage ccRCC (n=40) with Progression Free Survival as endpoint

Variables	Univariate analysis	Multivariate analysis		
ccRCC	*p*-value	HR	95 % CI	*p*-value
CK2α Nuc+ expression	0.043*	5.19	0.64-42.3	0.124
Fuhrman (G3+G4)	0.03*	4.79	1.41-16.28	0.012*
Tumorsize ≥ 7 cm	0.21	2.57	0.81-8.2	0.111
Female	0.07	3.42	1.05-11.07	0.04*
VHL mutation	0.64	1.24	0.45-3.45	0.68

* p <0.05

### Western blotting and CK2 kinase activity

**Figure 5:**
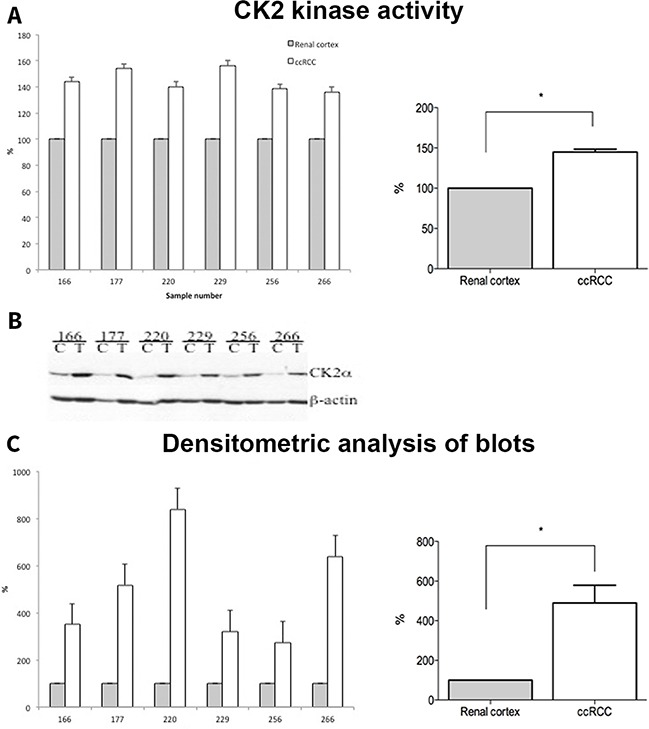
**A.** Whole cell lysates from normal (Renal cortex) and tumor (ccRCC) tissue samples (30 μg) were subjected to CK2 kinase activity assay as described in the materials and methods. The activity is expressed as percentage of control (Renal cortex). **B.** 30 μg of whole cell lysates from control (C, renal cortex) and tumor (T, ccRCC) were subjected to SDS-polyacrylamide gel electrophoresis (PAGE). Separated proteins were transferred to polyvinylidene difluoride (PVDF) membrane by western blot. Proteins were visualized by probing the membranes with antibodies against CK2α and β-actin, respectively. **C.** Densitometric analysis of protein bands is expressed as percentage of control.

Kinase activity of CK2 was significantly higher in the ccRCC samples compared to normal renal cortex, *p* = 0.03 (Figure 5A). To validate the IHC results, we performed an analysis of CK2α protein expression by Western blotting in a subset of 6 ccRCC samples together with normal renal cortex. As shown in Figure 5B-5C, CK2α protein expression was significantly higher in the tumor tissue compared to normal renal cortex.

### Proliferation assay

**Figure 6:**
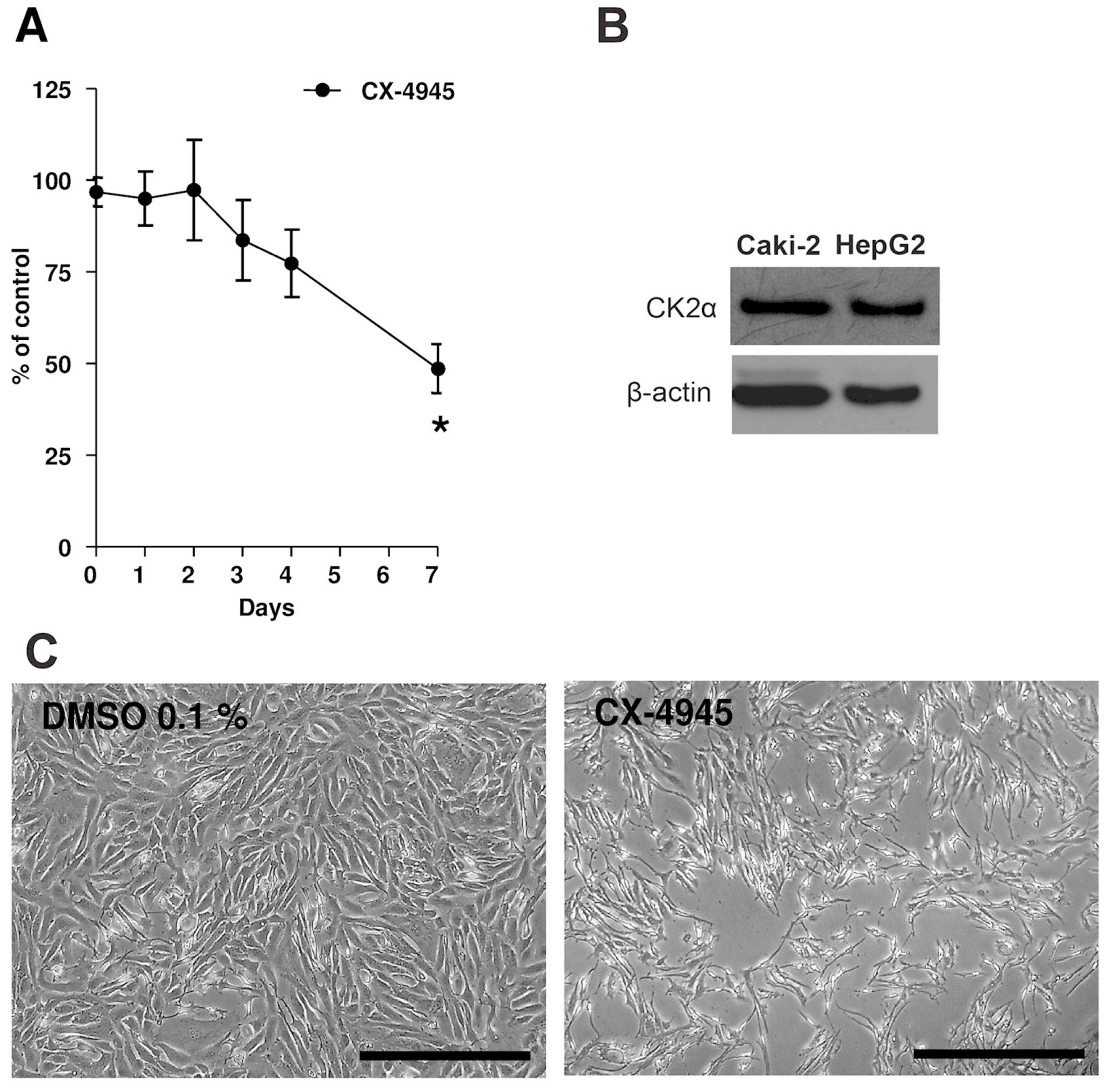
**A.** Caki-2 cells were treated with CX-4945 (10 μM) for 7 days. Experiments were repeated three times and data (absorption, ABS) were expressed as the means ± SEM of 3 replicates for each condition. Absorbance values were normalized to vehicle (DMSO). Student's T-test was used for statistical comparison of data sets at any given time point. *p< 0.01 vs. Control (vehicle). **B.** Western blot analyses of CK2α in Caki-2 cell lysates and HepG2 not treated with CX-4945. HepG2 served as a positive control. Actin expression served as a loading control. **C.** Pictures showing Caki-2 cells at the seventh day of the proliferation assay. At day 7, vehicle (DMSO 0.1%) was confluent, while cells in the presence of CX-4945 (10 μM, right picture) show a reduction to 49%. The scale bar in each picture corresponds to 500 μm.

In order to investigate whether CK2α could also be a therapeutic target of ccRCC, we evaluated the effects of two CK2 inhibitors (CX-4945 10 μM and E9 50 μM) on proliferation of Caki-2 cells using a colorimetric assay. Cells proliferated in a time-dependent fashion regardless of the treatment. However, CX-4945 significantly inhibited the proliferation of Caki-2 cells compared to vehicle-treated controls (Figure 6A and 6C, 49 % of control at confluence (*indicates p<0.01 vs. vehicle)). Figure 6B shows protein expression of CK2α by Western blot analysis in Caki-2 lysates and HepG2 cells not treated with CX-4945. E9 did not inhibit Caki-2 cell proliferation (Supplementary Figure S1).

## DISCUSSION

In a study done previously by our group, we discovered that high CK2α mRNA expression correlated with poor overall survival, disease specific survival and progression free survival in patients with ccRCC [24]. In this study our aim was to investigate the impact of a high protein expression of CK2α on survival of patients with ccRCC and furthermore, mRNA and protein expression of CK2 in the different subtypes of RCC and oncocytoma.

Protein kinase CK2α is one of the two isoforms of the catalytic subunit of the protein kinase CK2 and is overexpressed in various malignancies. Previously it has been shown that protein kinase CK2 activity in kidney cancer is higher compared to normal renal cortex [14], but the association between CK2α and clinicopathological features or prognosis in RCC remains unknown. In the present study, we assessed mRNA expression of CK2α, α' and β subunits and protein expression levels of CK2α subunit in different subtypes of RCC (clear cell, papillary and chromophobic) and in the benign renal tumor, oncocytoma. Messenger RNA expression of all CK2 subunits was significantly up-regulated in tumor tissue of ccRCC compared to renal cortex. In addition, high mRNA expression of CK2α correlated with poor prognostic markers (high Fuhrman grade, high stage and late metastasis), whereas high mRNA expression of CK2α' correlated with good prognosis. At protein level, a positive CK2α staining in the nuclei was associated with poor PFS in the patient group with high stage cancer. We found a higher protein expression in the ccRCC tissue compared to normal renal cortex by western blotting and in addition, a higher kinase activity in the tumor.

The catalytic subunits of CK2 are known to exert a significant protective effect against apoptosis, whereas the β-subunit does not induce a comparable effect [25]. The subunits of CK2 can exist in a dissociated form in the cell and in different compartments. Hence, their localization in specific cellular regions might represent a mode of functional regulation of CK2 that affects cell survival [26]. Our results indicate that CK2α is highly expressed at both mRNA and protein levels. The protein is mainly localized in the nucleus and its expression has a negative impact on prognosis. Findings reported here, support the notion that the individual CK2 subunits might exert functions independent from the holoenzyme as previously reported.

Laramas et al provided evidence for a strong association between nuclear expression of CK2α and poor prognostic factors in human prostate cancer [19]. However, they did not show any correlation between cytoplasmic staining and poor prognosis. Our findings are consistent with this study, in which only the nuclear overexpression of CK2α was found to have an adverse effect on prognosis. In a minor study by Roelants et al, the expression and enzymatic activity of the CK2 subunits were investigated in 15 patients with RCC [27]. They could show that the CK2α and α' subunits were overexpressed at protein level and it correlated with an enhanced CK2 kinase activity. These findings are very similar to the findings of our study. However, at mRNA level, a strong downregulation of these subunit transcripts in tumor samples compared with normal tissues was found, contradictory to what we discovered in our mRNA studies. There could be several reasons for this discrepancy. First of all, we included more than 100 patients with the ccRCC subtype and second of all, we used other reference genes for normalization. They furthermore analyzed the importance of CK2 activity on the proliferation of renal cancer cells in 786-O RCC cells (786-O WT) or isogenic 786-O cells either reconstituted with functional VHL (786-VHL) or expressing the empty expression vector pBABE (786-O pB) and exposed them for increasing concentrations of CX-4945. The growth of all three cell lines was inhibited in a concentration-dependent fashion. This is in accordance with the results obtained in our study.

The basic mechanisms behind the association between an unfavorable prognosis and high CK2 expression are still being explored. CK2 regulates multiple pathways including PI3K/Akt and WNT signaling cascades, NF-κB transcription, and the DNA damage response [12, 28]. Several functions of CK2 have been identified through which high CK2 activity favors neoplastic growth; it enhances the transforming potential of oncogenes [29], it supports neovascularization [30], it potentiates the multi-drug resistance phenotype [31], and it generates abnormal pro-survival and anti-apoptotic signals [32]. In addition, it has been shown that inhibition or down-regulation of CK2 leads to apoptosis in several cancer cell lines [33–36]. CK2 has been shown to regulate PI3K/Akt signaling at multiple junctions along the signaling cascade including the Akt protein [12]. PI3K/Akt signaling is known to regulate the response of endothelial cells to growth factor signaling including proliferation, migration and tube formation [37] and represents thereby a molecular mechanism through, which CK2 can drive angiogenesis. Another mechanism by which CK2 may contribute to angiogenesis is through regulation of HIF-1α. HIF-1α is often highly active in renal cell carcinoma due to mutations in VHL and subsequently accumulation of HIF-1α. Hypoxia increases the activity of CK2 and elevated CK2 activity results in the up-regulation of HDAC1/HDAC2 histone deacetylases that inhibit the expression of tumor suppressor genes pVHL and p53, thereby increasing HIF-1α [38]. HSP-90 regulates a VHL–independent HIF-1α degradation pathway. During hypoxia, HIF-1α associates with HSP90 and becomes stabilized. HSP90 and its co-chaperone Cdc37 are also substrates for protein kinase CK2 [39]. A comprehensive study investigating the signaling pathways of CK2 in renal cancer was beyond the scope of this study. However, Roelants et al did show that CX-4945 inhibited phosphorylation of AKT, p21 and α-catenin in renal cancer cell lines and moreover, they could show that CX-4945-induced p38MAPK activation correlated with increased p53 expression [27].

It has also been shown that an inverse relationship between promyelotic leukemia protein (PML) and CK2 activity exists and that PML degradation upon CK2 activation can account for the frequent loss of PML expression observed in multiple human tumors [12]. PML is a nuclear-matrix-associated protein and specifically a nuclear accumulation of CK2α could therefore be functionally relevant in order to inactivate the tumor-suppressive functions of PML [19].

In this study, the relationship between both a high mRNA expression and a positive nuclear expression of CK2α and distant metastasis suggested that increased expression of CK2α might accelerate the migration and invasion of tumor cells. To illustrate the potential mechanisms of CK2α in proliferation of tumor cells, we performed in vitro functional studies testing two different selective inhibitors of CK2 in predetermined concentrations [38, 40, 41].

We investigated the impact of CX-4945 and E9 on Caki-2 cell growth. The CK2 inhibitor CX-4945 exhibits anticancer activity by down-regulation of PI3K/Akt, p21 and HIF-1α and by that affects the transcriptional regulation of factors involved in cancer proliferation, angiogenesis and pro-inflammatory cytokine production. CK2 inhibition results in apoptotic cell death in tumor cells [42]. We also tested E9, which is a novel highly selective CK2 inhibitor. E9 leads to the degradation of HIF-1α during hypoxia and reduces the activation of ERK, which controls diverse cellular processes such as proliferation, survival and differentiation [38]. In our study we observed a significant reduced proliferation of the cells treated with CX-4945 compared to vehicle (reduced to 49% of control), whereas the cells treated with E9 grew in almost same density as vehicle. In a study by Guerra et al the efficacy of E9 on inhibition of CK2 was investigated in several cell lines, including a human renal carcinoma cell line (Cal54) [38]. They could show that some cell lines were more prone to E9 inhibition in comparison to others. E9 inhibited Cal54 and PC3 (human prostate cancer) to a less extent than Wit49 (Wilm's tumor), HepG2 (human hepatoma), Panc-1 (human pancreatic carcinoma) and Mia-Paca-2 (human pancreatic carcinoma). Also PARP cleavage analysis by Western blotting investigating the induction of apoptosis by E9 showed significant differences among the cell lines with no significant PARP cleavage detected in Cal54, Wit49 and PC3 cells. The different response in cell proliferation by treatment with CX-4945 and E9 in our study might be caused by distinct and/or unrelated intracellular targets of the two inhibitors. An unevenly CK2 inhibition by the chosen concentrations of CX-4945 and E9 could be another reason.

In conclusion, our study revealed up-regulated CK2α, α' and β expression levels in ccRCC tumor tissue compared to normal renal cortex, but only high expression of CK2α was associated to poor prognostic factors (high stage, high Fuhrman grade and distant metastasis). At protein level, nuclear localization of CK2α correlated to a poor PFS and to distant metastasis suggesting that CK2α contributes to migration and invasion of tumor cells. Proliferation studies revealed an inhibitory effect of CX-4945 on Caki-2 cell growth. Thus, inhibition of CK2 is a promising approach for ccRCC treatment in combination with other treatment modalities. All together, our results indicate that CK2α may serve as a candidate prognostic biomarker and a new therapeutic molecular target for ccRCC.

## MATERIALS AND METHODS

### Inclusion of patients

Two different patient cohorts were included in this study. A cohort for the purpose of generating quantitative RT-PCR data (n = 139) and a larger cohort for the purpose of performing immunohistochemistry (n=155), consisting of the patients included in the first cohort and 16 additional patients. The first cohort consisted of: ccRCC (n=97), also included in a previous study done by our group [24] and additionally; PRCC (n=23), ChRCC (n=8) and RO (n=11). The second cohort consisted of: ccRCC (n=105), PRCC (n=27), ChRCC (n=8), RO (n=13) and unclassifiable RCC (UcRCC, n=2).

### Patient characteristics

We obtained frozen tumor tissue and normal renal cortex from 139 patients and paraffin-embedded tumor specimen and control tissue (healthy renal cortex) from the same 139 patients and additional 16 patients being nephrectomized because of either RCC or oncocytoma at the Odense University Hospital, Denmark, between 2001-2012. Prior patient consent and approval from the Danish Ethics Committee (notification number 29573, permit no. S-VF-20010035, Region of Southern Denmark) and the Danish Data Protection Agency (file number 13/14405, permit no. 2008-58-0035, Odense University Hospital) were obtained. Information concerning the date of initial diagnosis, clinical characteristics, relapse/metastasis and death were obtained retrospectively. The patients were followed up until death, last contact or censored on November 2015.

### qRT-PCR

Quantitative RT-PCR data for the CK2 subunits, α, α' and β (gene symbols CSNK2A1, CSNK2A2 and CSNK2B respectively) from 97 ccRCCs were generated using a Taqman Array and analyzed as described in detail in a previous study carried out by us [24]. See Table 5 for details on assay ID and gene reference sequence.

**Table 5:** Genes used in qRT-PCR (Taqman Low Density Array). * indicates genes used as reference genes for normalization

Gene symbol	Gene name (Assay ID)	NCBI RefSeq	Gene function	Assay location (amplicon size)
CSNK2A1	Casein kinase 2, alpha 1 polypeptide (Hs00601957_m1)	NM_177559.2	Serine/threonine protein kinase	169(118)
CSNK2A2	Casein kinase 2, alpha prime polypeptide (Hs00176505_m1)	NM_001896.2	Catalytic subunit alpha' gene of human protein kinase CK2	1213(63)
CSNK2B	Casein kinase 2, beta polypeptide (Hs00365835_m1)	NM_001320.5	Beta subunit of human protein kinase CK2	413 (83)
HMBS*	Hydroxymethylbilane synthase (Hs00609297_m1)	NM_000190.3 NM_001258208.1	Enzyme of heme biosynthetic pathway	186 (64) 186(64)
TBP*	TATA box binding protein (Hs00427621_m1)	NM_001172085.1 NM_003194.4	Modulates DNA binding activity	666(65) 868(65)
PPIA*	Peptidylprolyl isomerase A (Hs99999904_m1)	NM_021130.3	Cyclosporin binding-protein	433(98)

In brief, total RNA from frozen tumor and normal renal cortex samples was isolated using the TRIZOL reagent (Invitrogen, United Kingdom). First strand cDNA synthesis was carried out using the iScript cDNA Synthesis Kit (Bio-Rad, CA, USA). The Applied Biosystems TaqMan Array (custom-designed 384-well micro fluidic card with TaqMan Gene Expression Assay) was used to conduct the qRT-PCR study. All qRT-PCR were run in duplicate on an ABI PRISM 7900 HT Sequence detecting system (Applied Biosystems, Foster City, CA, USA). Tumor and cortex samples from the same patient were always run on the same plate.

qRT-PCR data were collected and evaluated with SDS 2.4 software (Applied Biosystems) and qBasePlus software (Biogazelle, Zwijnaarde, Belgium). TBP, PPIA and HMBS were used as reference genes and normalization was done using the qBasePlus software according to the modified Δ ΔCT method for multiple reference genes [43, 44].

### Preparation of whole cell lysate, Western blot analysis and antibodies

Human frozen tissue samples from ccRCC and normal renal cortex (n=6) were re-suspended in lysis buffer [50mM Tris/HCl (pH 7.5), 150mM NaCl, 1% Triton X-100, 10% glycerol, 1mM DTT, 1 mM Na3VO4, 30 mM β-glycerophosphate, 10 mM NaF, 100 nM okadaic acid] containing a protease inhibitor cocktail (Roche, Switzlerland) and further processed as described elsewhere [45, 46]. The catalytic α-subunit of protein kinase CK2 was detected by probing the Western blot membrane with affinity-purified rabbit polyclonal anti-CK2α antibody obtained by immunizing rabbits with the full-length protein. Equal protein loading was verified by re-probing the Western blot membrane with a mouse monoclonal anti-β-actin antibody (Sigma-Aldrich, Missouri 63103, USA). Densitometric analysis of protein bands was performed with the Image J software.

### Construction of Tissue Micro Arrays

The procedure for construction of TMAs was as follows: representative areas of the different lesions were carefully selected on H&E stained sections under the light microscope and marked on individual paraffin blocks. Cores of 3.0 mm were punched from the selected contributive paraffin blocks and distributed into 26 new blocks including three cores from each tumor and one core from each normal renal cortex parenchyma.

### Immunohistochemistry

The tissue microarrays were used for immunohistochemical staining of CK2α. Immuno histochemical staining was performed using Dako Powervision (Dako, Denmark A/S) detection system on an AutostainerPlus (Dako, Denmark). Briefly, sections of 3 μm were cut on a microtome and mounted on slides. After deparaffination in xylene, slides were driven through different grades of ethanol to water, treated with 1.5% H_2_O_2_ for 10 min to block the endogenous peroxidase activity and heated for antigen retrieval in TRS buffer (Dako, Denmark A/S) for 15 min. The slides were then incubated with the primary rabbit CK2α polyclonal antibody for 60 min at a 1:400 dilution and incubated with the secondary antibody Poly-HRP anti-mouse/rabbit IgG (Novocastra PowerVision+Poly-HRP IHC Detection Systems, Leica Biosystems) for 30 min. Sections were counterstained with Mayer's hematoxylin and visualized with diaminobenzidine (DAB+ chromogen, DAKO Denmark, A/S). Slides were afterwards mounted automatically with cover films. As positive controls we used a multiblock containing different normal tissues, among those normal colon, testis and placenta, which were used as controls in this study. The negative control consisted of omission of the primary antibody.

### Evaluation of staining

The stained TMAs were scored separately by two pathologists (N.M. and M.R.) in a blinded fashion. Semi-quantitative assessment of antibody staining was performed using the following score: the extent of staining of moderate to strong intensity in the nuclei was scored as 0 (0%), 1+ (1-25%), 2+(26-50%), 3+(51-75%) or 4+(76-100%) according to the percentages of the positive staining areas relative to the entire core area. The same scoring has been previously evaluated and found feasible in previous studies on CK2 [16, 47, 48]. The cytoplasm was scored as 0 or 1+, when more than 10% of the cytoplasm area was positive. A mean score for cytoplasm and nuclei was obtained for the three cores from the same patient. The sum of scores for nuclei and cytoplasm was used as the final CK2α score (0-5+). For statistical purposes, tumors with a final staining score of >2+ were considered strongly positive for CK2α.

### Assessment of VHL mutations

Tissue microarrays were stained for VHL using an Autostainer (BenchMark Ultra, Ventana Medical System, Tucson, AZ, USA). Sections were blocked for endogenous peroxidase activity using H_2_O_2_ and treated enzymatically with protease 1 for 4 min (760-2018, Ventana Medical System, Tucson, AZ, USA) for epitope retrieval. The TMAs were then incubated for 32 min at 36°C with the primary antibody against VHL (SC-5575, Santa Cruz Biotechnology, TX, USA) at a dilution of 1:400. Detection and visualization was carried out using OptiView-DAB (Ventana Medical System, Tucson, AZ, USA). Sections were finally counterstained with hematoxylin (Ventana Medical System, Tucson, AZ, USA).

For analysis of expression of VHL, data were distributed into two categories: normal (> 10%) vs. altered (≤10%) according to the method described by Weber et al [49].

### Protein kinase assay

The kinase activity of CK2 was performed as previously described [50] using a CK2-specific synthetic peptide substrate RRRADDSDDDDD and 30 μg whole cell lysate.

### Cell culture

A cell line established from a primary clear cell carcinoma of the kidney, Caki-2 (HTB-47, commercially available from American Type Culture Collection (ATCC), Rockville, MD) was cultured in McCoy's 5A medium (ATCC, Rockville, MD), supplemented with L-glutamine, 10% Foetal Bovine Serum (Gibco, Thermo-Fisher, Waltham, MA USA) and 1% penicillin/streptomycin (Sigma-Aldrich, MO, USA). Cells were cultured continuously in a humidified 5% CO_2_ incubator at 37°C.

### Cell proliferation assay

Cell proliferation was spectrophotometrically assessed as described previously [51, 52] with some modifications. Briefly, Caki-2 cells were seeded at the same density (80.000 cells/well) in 12-well plates (flat bottom, Costar, Corning Inc. NY, USA) and cultured in McCoy's 5A medium supplemented with L-glutamine, with 10% foetal bovine serum and 1% penicillin/streptomycin, in the presence of CX-4945 (10 μM), E9 (50 μM) or vehicle (DMSO 0.1% for CX-4945 or DMSO 0.5% for E9). Final DMSO concentrations were the same for each compound and its control. Sterile filtered foetal bovine serum served as standard mitogenic stimulus. Cells were fixed with formalin (10% in deionized water) at days 0,1,2,3,4, and 7 (confluence). Thereafter, cells were stained for 10 min with 0.3% Janus B Green dye at room temperature under constant stirring. Cells were then de-stained with water and dye was eluted with 0.5 M HCl at room temperature under constant stirring for 15 min. Absorbance at 595 nm was determined using a microplate reader (Sinergy HT, Biotek, USA). Absorbance values correlated with cell density. We performed three independent experiments.

### Statistical analysis

Comparisons between tumor and renal cortex from the same patient were made using the non-parametric Wilcoxon signed rank test. Kruskal-Wallis test followed by Dunn's multiple comparisons test was used for analyzing differences between more than two groups. Associations between the categorical variables were assessed by means of the χ^2^ tests. A p-value <0.05 was considered statistically significant. OS (overall survival) was defined as time from the date of imaging diagnosis to the date of death from any cause or last follow-up contact at any hospital department of urology or oncology. DSS (disease specific survival) was defined as time from the date of imaging diagnosis to the date of death from RCC or last follow-up contact. Progression free survival (PFS) was calculated from the date of diagnosis by imaging to the date of progression, death from any cause or last follow-up contact. Late metastasis was defined as metastasis at the time of follow-up. OS, DSS and PFS in 40 patients with high stage (pT3-pT4) ccRCC was estimated by the Kaplan Meier method and assessed by the log-rank test. Univariate/multivariate Cox regression analyses were applied on the same patient group. Statistical analysis was carried out using 14.0 STATA software (StataCorp, Texas, USA).

## SUPPLEMENTARY FIGURE


