# Supplementary material for: Nuclear localization of the CK2α-subunit correlates with poor prognosis in clear cell renal cell carcinoma
Source: Oncotarget. 2016 Nov 29;8(1):1613–27. doi: 10.18632/oncotarget.13693 (PMC5352082; doi:10.18632/oncotarget.13693)
Supplement: Supplementary file 1 [file oncotarget-08-1613-s001.pdf]

Nuclear localization of the CK2α-subunit correlates with poor prognosis in clear cell renal cell carcinoma

SUPPLEMENTARY FIGURE

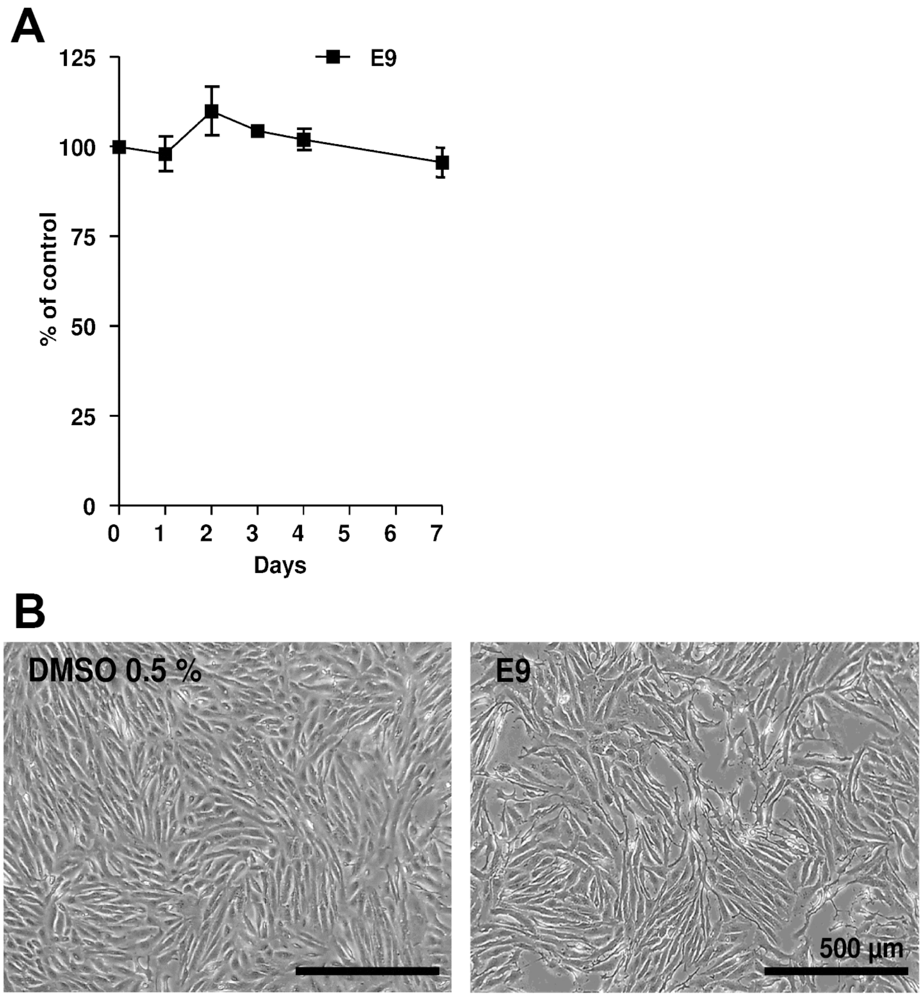

Supplementary Figure S1: A. Caki-2 cells were treated with the CK2 inhibitor E9 (50 µM) for 7 days. Experiments were repeated three times and data (absorption, ABS) were expressed as the means ± SEM of 3 replicates for each condition. Absorbance values were normalized to vehicle (DMSO). Student's T-test was used for statistical comparison of data sets at any given time point. No significant difference was found in the proliferation of the cells by treatment with E9 compared to vehicle **B.** Pictures showing Caki-2 cells at the seventh day of the proliferation assay. At day 7, the control (DMSO 0.5%) was confluent (left picture). No significant reduction in cell number was seen when cells were treated with E9 (right picture). The scale bar in each picture corresponds to 500 µm.
